# Author Correction: Physical activity and screen time of children and adolescents before and during the COVID-19 lockdown in Germany: a natural experiment

**DOI:** 10.1038/s41598-021-03905-5

**Published:** 2021-12-15

**Authors:** Steffen C. E. Schmidt, Bastian Anedda, Alexander Burchartz, Ana Eichsteller, Simon Kolb, Carina Nigg, Claudia Niessner, Doris Oriwol, Annette Worth, Alexander Woll

**Affiliations:** 1 Karlsruhe Institute of Technology, Engler-Bunte-Ring 15, 76131 Karlsruhe, Germany; 2 University of Education Karlsruhe, Bismarckstraße 10, 76133 Karlsruhe, Germany

Correction to: *Scientific Reports* https://doi.org/10.1038/s41598-020-78438-4, published online 11 December 2020

The original version of this Article contained an error in Reference 29, which was incorrectly given as:

Chen, P. *et al.* Wuhan coronavirus (2019-nCoV): the need to maintain regular physical activity while taking precautions. *J. Sport Health Sci.* **9**(103–104), 2020. https://doi.org/10.1016/j.jshs.2020.02.001 (2020).

The correct reference is listed below:

Chen, P. *et al.* Coronavirus disease (COVID-19): The need to maintain regular physical activity while taking precautions. *J. Sport Health Sci.* **9**(103–104), 2020. https://doi.org/10.1016/j.jshs.2020.02.001 (2020).

The original Article has been corrected.

